# Observation of the effect of bone marrow mesenchymal stem cell transplantation by different interventions on cirrhotic rats

**DOI:** 10.1590/1414-431X20187879

**Published:** 2019-02-25

**Authors:** Xiaoling Zhou, Jianqing Yang, Ying Liu, Zepeng Li, Jingfang Yu, Wanhua Wei, Qiao Chen, Can Li, Nong Tang

**Affiliations:** 1 Graduate School of Hunan University of Traditional Chinese Medicine, Changsha, Hunan, China; 2 Department of Gastroenterology, Liuzhou Traditional Chinese Medicine Hospital, Liuzhou, Guangxi, China; 3 Department of Surgery, Liuzhou Traditional Chinese Medicine Hospital, Liuzhou, Guangxi, China; 4 Guangxi University of Traditional Chinese Medicine, Nanning, Guangxi, China

**Keywords:** Bone marrow mesenchymal stem cells, Transplantation, Liver cirrhosis, Granulocyte colony-stimulating factor, Jisheng Shenqi decoction

## Abstract

Bone marrow mesenchymal stem cells (BMSCs) transplantation has attracted attention for the treatment of liver cirrhosis and end-stage liver diseases. Therefore, in this study, we evaluated the effect of different methods of BMSCs transplantation in the treatment of liver cirrhosis in rats. Seventy-two male Sprague-Dawley rats were divided into 7 groups: 10 were used to extract BMSCs, 10 were used as normal group, and the remaining 52 rats were randomly divided into five groups for testing: control group, BMSCs group, BMSCs+granulocyte colony-stimulating factor (G-CSF) group, and BMSCs+Jisheng Shenqi decoction (JSSQ) group. After the end of the intervention course, liver tissue sections of rats were subjected to hematoxylin and eosin (H&E) and Masson staining, and pathological grades were scored. Liver function [aminotransferase (ALT), aspartate aminotransferase (AST), albumin (ALB)] and hepatic fibrosis markers [hyaluronidase (HA), laminin (LN), type III procollagen (PCIII), type IV collagen (CIV)] were measured. BMSCs+JSSQ group had the best effect of reducing ALT and increasing ALB after intervention therapy (P<0.05). The reducing pathological scores and LN, PCIII, CIV of BMSCs+G-CSF group and BMSCs+JSSQ group after intervention therapy were significant, but there was no significant difference between the two groups (P>0.05). The effect of JSSQ on improving stem cell transplantation in rats with liver cirrhosis was confirmed. JSSQ combined with BMSCs could significantly improve liver function and liver pathology scores of rats with liver cirrhosis.

## Introduction

Cirrhosis is a consequence of chronic liver disease characterized by replacement of liver tissue by fibrotic scar tissue as well as regenerative nodules, leading to progressive loss of liver function (1). Incidence of liver cirrhosis is rising worldwide with expected increases in hospital admissions and cirrhosis-related deaths (2). The incidence of liver cirrhosis in China is increasing yearly, and about one million people die from liver cirrhosis each year, which is a serious public health problem (3).

Many studies have shown evidence that transplantation of bone marrow mesenchymal stem cells (BMSCs) can sustain liver function after liver damage (4). An *in vitro* study has shown that BMSCs induce apoptosis and suppress collagen synthesis in hepatic stellate cells (5). Additionally, *in vivo* studies have confirmed that BMSCs injected through a peripheral vein have antifibrotic and anti-inflammatory functions (6,7). The main problem affecting the efficacy of BMSCs transplantation in the treatment of liver cirrhosis is that the number of BMSCs homing to the injured liver after transplantation is insufficient. Therefore, a safe and effective stem cell mobilizer is the key to improve the efficacy of BMSCs transplantation.

Granulocyte colony-stimulating factor (G-CSF) is an effective stem cell mobilizer, but its comprehensive curative effect on liver cirrhosis patients is limited, and long-term use costs are high (8,9). Some studies have suggested that traditional Chinese medicine could promote the function of bone marrow regeneration and promote the activation, migration, proliferation, and differentiation of BMSCs (10,11).

This study intended to use different methods of BMSCs transplantation in a cirrhosis rat model, and observe the changes of liver function, fibrosis, and histopathology, and other related indicators before and after intervention, providing experimental evidence for end-stage liver disease treated by convenient and efficient stem cell mobilizers.

## Material and methods

### Animals, reagents, and drugs

A total of 72 male Sprague-Dawley rats (SD; weight 200±20 g), of which 10 were used to extract BMSCs and 10 were fed until the end of the experiment from which liver and abdominal aortic blood were taken after sacrifice for indicators as the normal group. The remaining 52 rats were used for the liver cirrhosis model and randomly divided into five groups for testing.

The animals were provided by the Experimental Animal Center of Guangxi University of Traditional Chinese Medicine with animal certification No. 11004 of Gui Medical Animal. This study was approved by Ethics Committee of Guangxi University of Chinese Medicine.

#### Reagents

Low-glucose DMEM medium (Dulbecco's modified Eagle medium, nutrient mixture F-12, DMEM/F12), phosphate buffered saline (PBS), fetal bovine serum, and trypsin were purchased from GIBCO (Grand Island Biologial Company, USA). CD34+ (catalog No. ab152203), CD44+ (catalog No. ab371437), and CD105+ (catalog No. ab120407) were purchased from BioLegend (China). CCl4 (99.5% purity) was purchased from UNI-CHEM Chemical Reagent (China). Olive oil, cyclophosphamide, 4% formaldehyde solution, and G-CSF were purchased from Kirin Kunpeng Biological Pharmaceutical Co., Ltd. (China). Liver function test kit was purchased from Sclavo (Italy) and liver fibrosis test kit was provided by China Atomic Energy Research Institute.

Jisheng Shenqi decoction (JSSQ) was composed of 80 g *Radix rehmanniae praeparata*, 40 g *Cornus officinalis*, 40 g Chinese yam, 30 g *Rhizoma alismatis*, 30 g *Poria cocos*, 30 g *Cortex moutan*, 10 g cinnamon, 10 g *Radix aconiti carmichaeli*, 20 g *Semen plantaginis*, and 20 g *Radix achyranthis*. These traditional Chinese medicines were decocted by Sanyan Chinese Herbal Boiler from Tianjin Sanyan Company, which complied with the relevant provisions of the 2010 edition of the Pharmacopoeia and was prepared by Liuzhou Traditional Chinese Medicine Hospital. The above compound was decocted at 100°C for 20 min, and decocted with water twice at 80°C for 30 min to remove the slag. The two-time decoction was blended and then concentrated in a Chinese medicine liquid packaging machine to contain a 3 g/mL crude drug solution as an intragastric dose, and was stored at 4°C in a refrigerator for future use.

### Isolation of stem cell, model preparation, and administration route

#### Isolation and culture of rat BMSCs

After one week of adaptive feeding, 10 healthy SPF-grade SD male rats were sacrificed by intraperitoneal injection of 10% chloral hydrate (10 mL). The specific steps were done as previously described (12). The femur and tibia were aseptically isolated and the cells in the bone marrow cavity were rinsed into the cell culture flask with L-DMEM medium containing 10% fetal bovine serum. The cells were incubated at 37°C with a volume fraction of 5% CO_2_, and the original culture medium was discarded the next day and replaced with a new one. In the culture medium, the adherent cells were bone marrow mesenchymal stem cells, which were passaged every 2 days and the third-generation cells were used for transplantation.

#### Identification of rat BMSCs

BMSCs were analyzed by fluorescence immunoassay to detect the surface markers (CD34, CD44, CD105). P3 cells were re-suspended in PBS for the immunophenotype analysis. BMSCs were stained with antibodies conjugated with phycoerythrin (PE): CD34-PE, CD44-PE, CD105-PE. The rat immunoglobulin IgG-PE was used as the control isotype at the same concentration as the specific primary antibodies. The cells were tagged for 45 min in the dark at room temperature, washed three times with PBS, and detected.

#### Rat model of liver cirrhosis (7)

Olive oil (50%) and CCL4 solution (1:1) was injected subcutaneously (3 mL/kg once a day for 3 days) into the abdomen of rats. The dose was adjusted according to the body weight of the rats. After 4 weeks of application, the body weight of the rats was observed to confirm that it was stable. If the weight was increased, the original amount was injected until the body weight was constant. At the 4th week, 2 rats were randomly selected and sacrificed. Liver biopsies were used to prepare liver tissue pathological sections to determine the success of liver cirrhosis. The remaining 50 successful liver cirrhosis rats were randomly divided into groups. The specific protocols are as previously described (13).

#### Grouping

The remaining 50 cirrhotic rats were randomly divided into groups as follows: normal group: 10 rats were fed until the end of the experiment, and liver and abdominal aortic blood were taken after sacrifice for indicators; control group: 10 liver cirrhosis rats were fed with equal amounts of saline daily, and were sacrificed at the end of 6 weeks and 12 h after fasting; BMSCs portal vein graft group (BMSCs group, n=10 rats): 1.5 mL solution containing 1.5×10^6^ of BMSCs was injected into the portal vein on the day of intervention, as a one-time treatment, and the animals were sacrificed on the 15th day after fasting for 12 h; BMSCs portal vein graft combined with G-CSF group (BMSCs+G-CSF group, n=10 rats): on the day of intervention, 1.5 mL solution containing 1.5×10^6^ of BMSCs was injected into the portal vein and 10 μg/kg G-CSF were subcutaneously injected as a one-time treatment, and the animals were sacrificed on the 15th day after fasting for 12 h; BMSCs portal vein graft combined with JSSQ group (BMSCs+JSSQ group, n=10 rats): 1.5 mL solution containing 1.5×10^6^ of BMSCs was injected into the portal vein on the day of intervention, 2 mL per day of JSSQ was intragastrically administrated at 9:00 am, for 14 days, and the animals were sacrificed on the 15th day after fasting for 12 h; JSSQ group (n=10 rats): 2 mL of JSSQ was intragastrically administrated at 9:00 am daily, for 14 days, and the animals were sacrificed on the 15th day after fasting for 12 h.

In addition to the above normal group, the rest of the treatment groups started from 4 weeks to the end of 6 weeks for a total of 2 weeks. One rat in the control group and one in the BMSCs+G-CSF group died during the experiment.

### Observation indexes and measurement standards

#### Detection of hepatic function and liver fiber

The rats were anesthetized and blood was taken from the abdominal aorta to measure serum alanine aminotransferase (ALT), aspartate aminotransferase (AST), hyaluronidase (HA), laminin (LN), type III procollagen (PCIII), and type IV collagen (CIV) levels using a Japanese Hitachi 7170S automatic biochemical analyzer.

#### Histopathological examination

After the end of treatment, all rats were sacrificed by intraperitoneal injection of 10% chloral hydrate. Twenty micrograms of liver tissue were weighed, frozen, and cut, fixed in 4% formaldehyde solution, embedded in paraffin, and stained with H&E and Masson. Under the light microscope (Olympus Corporation Japan), the histological grades of the two groups were recorded (10). The degree of hepatocyte necrosis was recorded as follows: score 0 was none, score 1 was little, score 2 was mild, score 3 was moderate, and score 4 was severe. Fibrosis grading was as follows: score 0 was normal, score 1 was increased collagen without gaps, score 2 was incomplete gaps, score 3 was fine complete gaps (false lobules), and score 4 was thick false leaflets. Fatty grade score was as follows: score 0 was no fat degeneration, score 1 was a small amount of fat-producing cells, score 2 was fatty degeneration ratio <1/3, score 3 was fatty degeneration ratio of 1/3–2/3, and score 4 was steatosis proportion ratio >2/3.

### Statistical analysis

The SPSS 17.0 statistical analysis software (USA) was used. Measured data are reported as means±SD, and count data are reported as ratio or constituent ratio. One-way ANOVA test was used to measure normal distribution data and LSD or SNK *post-hoc* tests were used for comparison between groups. Abnormal distribution data were tested with rank sum tests. Counting grade data were tested with rank sum tests. P<0.05 was considered statistically significant.

## Results

### Isolation, culture, and surface marker assays of rat BMSCs

**Figure 1:**
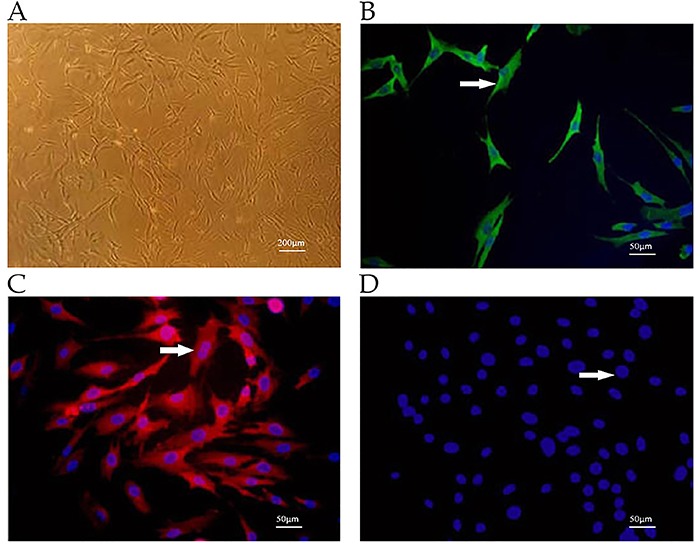
Expression of molecules on cells by fluorescence immunoassay (×400; A: 200 μm, B–D: 50 μm). Spindle-shaped inter-bone marrow stem cells (**A**), surface antigens included CD105+ (**B**), CD44+ (**C**), and CD34+ (**D**). Arrows indicate that isolated cells were bone marrow-derived mesenchymal stem cells.

As shown in Figure 1, cells from early isolation and culture were round and stretched, and spindle-shaped inter-bone marrow stem cells were significantly increased, scattered or clustered (Figure 1A). To identify the origin of these cells, we next detected the expression of BMSC markers CD34, CD44, and CD105 by fluorescence immunoassay. The results showed that the cell homogeneity was good, the positive rate of CD105+ was 88.5% (Figure 1B), the positive rate of CD44+ was 99.4% (Figure 1C), and the positive rate of CD34+ was 0.59% (Figure 1D), indicating that spindle cells were BMSCs.

### Liver function test

**Figure 2:**
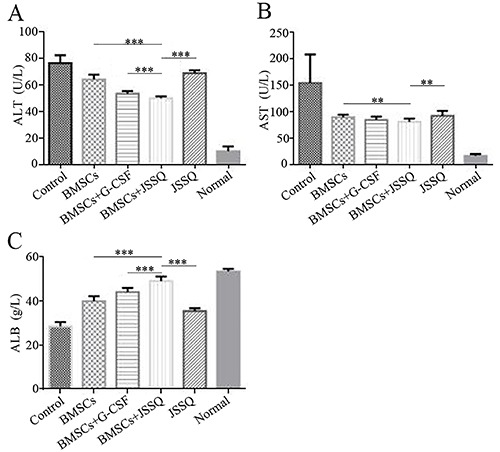
Effect of different methods of bone marrow-derived mesenchymal stem cells (BMSCs) transplantation in the treatment of liver cirrhosis in rats. G-CSF: granulocyte colony-stimulating factor; JSSQ: Jisheng Shenqi decoction. Levels of serum alanine aminotransferase (ALT) (**A**), aspartate aminotransferase (AST) (**B**), and albumin (ALB) (**C**) in liver function tests are shown. Data are reported as means±SD **P<0.01, ***P<0.001 (ANOVA).

As shown in Figure 2, ALT and AST of liver cirrhosis rats in BMSCs group, BMSCs+G-CSF group, BMSCs+JSSQ group, and JSSQ group showed different degrees of reduction after treatment (P<0.01). BMSCs+JSSQ group had the best effect of reducing ALT, which was significantly better than BMSCs+G-CSF group (P<0.05). BMSCs+G-CSF and BMSCs+JSSQ had no difference on reducing AST (P>0.05). BMSCs+JSSQ and BMSCs+G-CSF groups significantly increased ALB, but the effect of BMSCs+JSSQ on ALB increase was better than in the BMSCs+G-CSF group (P<0.01).

### Detection of hepatic fibrosis indicators

**Figure 3:**
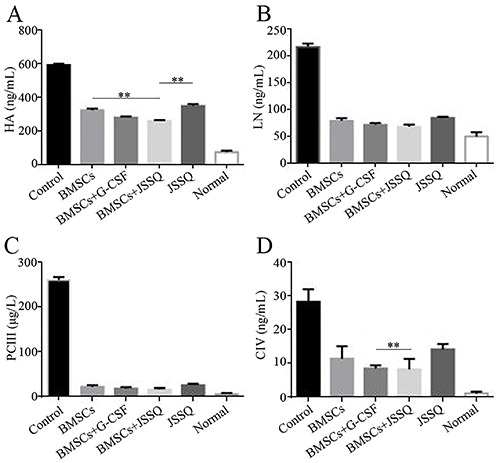
Detection of hepatic fibrosis after different methods of bone marrow-derived mesenchymal stem cells (BMSCs) transplantation in the treatment of liver cirrhosis in rats. G-CSF: granulocyte colony-stimulating factor; JSSQ: Jisheng Shenqi decoction. Levels of hyaluronidase (HA) (**A**), laminin (LN) (**B**), type III procollagen (PCIII) (**C**), and type IV collagen (CIV) (**D**) are shown. Data are reported as means±SD. **P<0.01 (ANOVA).

As shown in Figure 3, after intervention treatment, LN, PCIII, and CIV of BMSCs group, BMSCs+G-CSF group, BMSCs+JSSQ group, and JSSQ group all showed different degrees of reduction (P<0.0001). The reduction of HA in the BMSCs+JSSQ group was better than in the BMSCs+G-CSF and JSSQ groups (P<0.01). Comparing the BMSCs+JSSQ, BMSCs+G-CSF, and JSSQ groups, there was no difference in the reduction of LN and PCIII. The reduction of CIV in the BMSCs+JSSQ group was better than that of the BMSCs+G-CSF group (P<0.001).

### Histopathological changes and pathological scores

**Figure 4:**
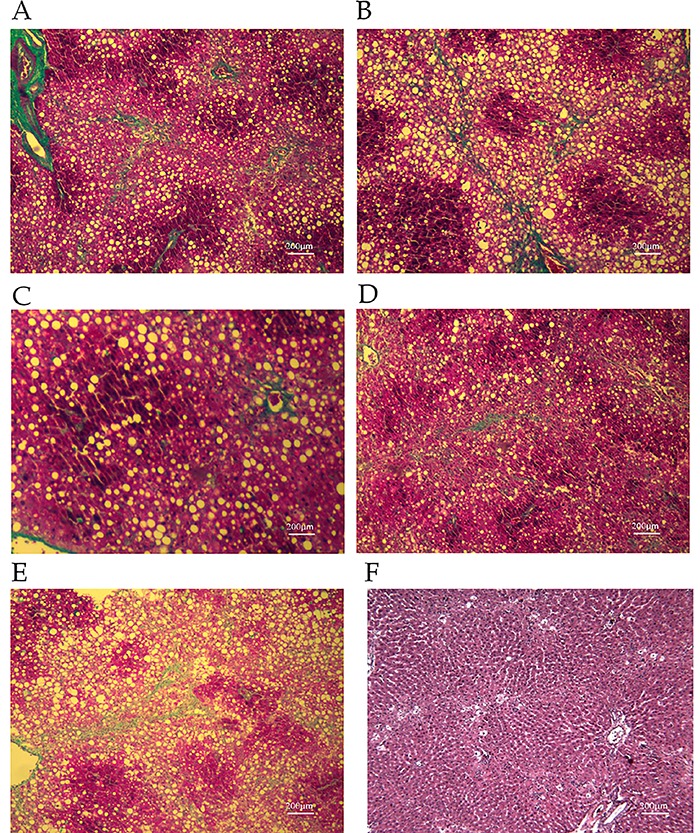
Pathological images of **A**, control (moderate and severe fibrosis, thick fibrous septae of the false lobule, heavy degree of hepatocyte degeneration and necrosis, and a lot of fat vacuoles); **B**, bone marrow mesenchymal stem cells (BMSCs); **C**, BMSCs+granulocyte colony-stimulating factor (G-CSF); **D**, BMSCs+Jisheng Shenqi decoction (JSSQ); **E**, JSSQ; **F**, normal groups (no obvious hepatic fibrosis, degeneration and necrosis of hepatocytes, and a little fatty degeneration) (H&E and Masson staining, bar 200 µm).

There was no hepatic fibrosis, degeneration, or necrosis of hepatocytes, and a little fatty degeneration was seen in the normal group (Figure 4F). In the liver tissue of the control group, moderate and severe fibrosis, thick fibrous septae of the false lobule, heavy degree of hepatocyte degeneration and necrosis, and many fat vacuoles could be seen under the microscope (Figure 4A). In the liver tissue of BMSCs, BMSCs+G-CSF, BMSCs+JSSQ, and JSSQ groups, moderate fibrosis was found, the fibrillary space of pseudo-lobule was slender, and the degree of hepatocyte degeneration and necrosis was lighter; only a small amount of fat vacuoles was seen (Figure 4B, C, D, E). Histopathological scores showed that the degree of hepatic fibrosis and hepatocyte degeneration and necrosis were lower in BMSCs, BMSCs+G-CSF, and BMSCs+JSSQ groups than in the control group (P<0.01). The degree of hepatic fibrosis and hepatocyte degeneration and necrosis in BMSCs+JSSQ and BMSCs+G-CSF groups were significantly reduced (P<0.01), but there was no significant difference in histopathological score between the two groups (P>0.05) (Table 1).

**Table 1:** Histopathological scores after treatment in cirrhotic rats.

Groups	Liver fibrosis	Liver cell degeneration and necrosis	Fatty degeneration	Total scores
Control	3.66±0.71*	3.88±0.33*	2.67±0.71*	9.56±0.53*
BMSCs	3.10±0.57*	3.10±0.32*	2.10±0.32*	9.0±0.94^#^
BMSCs+G-CSF	2.33±0.70*	2.78±0.44*	1.56±0.53*	6.89±0.60
BMSCs+JSSQ	2.20±0.63	2.50±0.53	1.40±0.52	6.30±0.67
JSSQ	3.50±0.53*	3.30±0.48*	2.30±0.48*	9.3±0.48^#^
	F (4, 43)=10.70	F (4, 43)=14.26	F (4, 43)=9.60	F (4, 43)=48.21
	*P<0.0001	*P<0.0001	*P<0.0001	*P<0.0001
				^#^P<0.001
Normal	0	0	0.30±0.48	0.30±0.48

Data are reported as means±SD. *^#^One-way ANOVA followed by paired LSD analysis was performed for comparison of all groups (except the normal group) with BMSCs+JSSQ group. BMSCs: bone marrow mesenchymal stem cells; G-CSF: granulocyte colony-stimulating factor; JSSQ: Jisheng Shenqi decoction.

## Discussion

China is a country with high incidence of hepatitis and cirrhosis. Nearly one million people worldwide die from cirrhosis and its complications each year, so finding a cost-effective treatment for cirrhosis is particularly important. In addition to drugs and orthotopic liver transplantation, BMSCs transplantation for the treatment of liver cirrhosis is a method that should be popularized.

Mesenchymal stem cells (MSCs) are stem cells with a high degree of self-renewal and multi-differentiation potential (14). They can proliferate and differentiate into a variety of functional cells, muscles, bones, and parts of internal organs. BMSCs are the earliest stem cells that can be differentiated into glial cells, neurons, stem cells, and other germ layers, and they have effects on self-proliferation, immune regulation, and repair of damaged organs (15–17). When the liver is damaged, BMSCs can home to the site of injury and differentiate and proliferate into hepatocytes, improving liver function and liver pathology scores (18,19).

ALT and AST mainly exist in liver cells, ALT in cytoplasm, and AST in mitochondria (20). When stem cells are injured by inflammation, ALT first enters the blood. When the cells are severely damaged and the mitochondria are compromised, AST will also enter the blood. It is known that ALT and AST are important indicators reflecting inflammation of the liver (21). The liver is an important site for the synthesis of ALB. When the liver is severely damaged beyond repair, the ability of the liver to synthesize ALB is significantly reduced.

Studies have proven that six-month liver function indexes are improved after an intravenous injection of cultured BMSCs, indicating the safety and effectiveness of BMSCs for treating cirrhosis (22 [Bibr B23]). In this study, it was found that BMSCs+JSSQ group had the best effect in reducing ALT and increasing ALB after intervention therapy, which was significantly better than the BMSCs+G-CSF group, indicating that BMSCs+JSSQ group was effective in cirrhotic rats. The hepatic inflammatory response and liver reserve function had significant improvement. This may be related to the fact that JSSQ induced more homing of BMSCs to the injured site, differentiated and proliferated stem cells, and promoted liver function repair.

HA, LN, PCIII, and CIV are commonly used indicators of liver fibrosis. Nowadays, serological examinations of HA, PCIII, LN, and CIV have become the most commonly used noninvasive method for detecting hepatic fibrosis (22–24). A direct relationship between hepatic fibrosis and these four serological indicators has been proven in many animal experiments and clinical studies.

Liver fibrosis is a necessary stage for the development of chronic hepatitis to liver cirrhosis. Effectively improving the patient's liver function, reducing the degree of liver fibrosis, thus delaying the further progress of patients with cirrhosis are the keys to clinical treatment of liver disease (25). PCIII is a precursor of type III collagen, reflecting the synthesis of fibrosis and inflammatory activity (26,27). The elevation of PCIII is closely related to the degree of hepatic fibrosis (28). As the degree of fibrosis increases, the level of PCIII may gradually increase. The study found that the effects in the BMSCs+G-CSF and BMSCs+JSSQ groups on the pathological scores of LN, PCIII, CIV, and liver cirrhosis after therapy were significant, with no significant difference between groups, indicating that the BMSCs+JSSQ group had a significant effect on hepatic fibrosis, hepatocyte steatosis, and inflammation necrosis in cirrhotic rats.

In this study, the effect of JSSQ on the improvement of stem cell transplantation in rats with liver cirrhosis was confirmed. This may be related to the fact that JSSQ exerted a similar cell-homing effect to G-CSF. As a class of inducer for cell homing, G-CSF has attracted the attention of many scholars (29,30). It has been experimentally confirmed that G-CSF could mobilize stem cells into the blood, prompt more stem cells to migrate to the injured liver, thereby participate in hepatocyte regeneration and repair (31,32). After treatment with BMSCs combined with JSSQ of liver cirrhosis in rats, the improvement of liver function, hepatic fibrosis, pathological tissue, and other related indicators was better than the combined G-CSF transplantation group (P<0.01). This indicated that JSSQ may play a role in mobilizing stem cells into the blood similar to G-CSF in stem cell transplantation, and its effect was even better than that of G-CSF. JSSQ has the function of nourishing liver and kidney, and promoting bone marrow regeneration, which had been widely used in clinical settings. Our previous study found that JSSQ combined with alpha-2b interferon showed a good curative effect on HBeAg positive chronic hepatitis B of spleen-kidney Yang deficiency (33). JSSQ could effectively promote BMSC homing to the liver after BMSC transplantation, and was safe and feasible (34).

At present, there are few experimental studies on the treatment of liver cirrhosis by Chinese medicine combined with BMSCs transplantation. Although G-CSF is a commonly used stem cell mobilizer, due to its relatively high cost, it is particularly important for us to consider the cost-effectiveness advantage of Chinese medicine for a similar replacement. In this study, after establishing a rat model of liver cirrhosis and intervening with BMSCs transplantation with different interventions, improvements of related indicators were observed, indicating that JSSQ combined with BMSCs could significantly improve liver function and liver pathology scores of rats with liver cirrhosis.
